# Surgery for pelvic organ prolapse and stress urinary incontinence and female sexual functions: A quasi-experimental study

**DOI:** 10.12669/pjms.37.4.3892

**Published:** 2021

**Authors:** Saida Abrar, Raheela Mohsin, Huda Saleem

**Affiliations:** 1 Dr. Saida Abrar, Clinical Fellow Urogynecology and pelvic reconstructive surgery, Department of Gynecology/Obstetrics, Aga Khan University Hospital, Karachi, Pakistan; 2 Dr. Raheela Mohsin Rizvi, Clinical Fellowship Urogynecology Sydney, Australia Associate Professor, OBGYN, section of Urogynecology, Aga Khan University Hospital, Karachi, Pakistan; 3 Dr. Huda Saleem, Resident Obstetrics/Gynaecology, Aga Khan University Hospital, Karachi, Pakistan

**Keywords:** Female sexual functions index, Pelvic organ prolapse, Stress urinary incontinence

## Abstract

**Objectives:**

To assess the effect of pelvic organ prolapse (POP) and/or stress urinary incontinence (SUI) on various domains of female sexual functions in patients before and after reconstructive surgery for these pelvic floor disorders.

**Methods:**

We conducted a quasi-experimental study of 126 women aged 25-65 years, presenting with POP / SUI, from January 1^st^ 2019 to December 31^st^ 2019 at Aga Khan University Hospital. POP surgery was performed only in patients with symptomatic POP ≥ stage 2 according to POP-Q (quantification). Sexual functions were assessed using Female Sexual Function Index (FSFI) questionnaire, among sexually active women at baseline and 18 months after surgery.

**Results:**

Mean age of the participants was 51.6, with a mean parity of four. Out of 126 patients, 31 patients underwent vaginal hysterectomy, pelvic floor repair and mid-urethral sling (MUS), 55 had vaginal hysterectomy with pelvic floor repair, 12 women had only pelvic floor repair and 10 patients had uterine suspension surgery for prolapse, while 18 patients underwent MUS operation alone for SUI. There was a statistically significant difference in female sexual functions after surgery for POP and/or SUI (p<0.01). This improvement was observed in both total and individual scores of each domain of FSFI with an overall improvement in sexual function from a mean of 18.5 pre-surgery to 20.8 post-surgery.

**Conclusions:**

This study reveals that women sexual functions are affected by POP and SUI and improve remarkably after reconstructive surgeries for these pelvic floor disorders.

## INTRODUCTION

Pelvic organ prolapse (POP) and stress urinary incontinence (SUI) is disorders of the pelvic floor with a significant impact on a woman’s quality of life.^1^ The global prevalence of POP and urinary incontinence is about 19.7% (range 3.4–56.4%) and 28.7% (range 5.2–70.8%) respectively.^2^ According to a UK study about 37 to 64% of women consulted the urogynecology clinic for problems of sexual dysfunctions.^3^ Sexual dysfunction reported with POP or SUI usually includes disorders of desire, arousal, orgasm, and dyspareunia. Owing to the close anatomical relationship and same pathophysiology, patients with prolapse may have coexistent urinary symptoms and vice versa.

Literature shows varying results regarding the effect of POP and SUI on sexual functions. While some studies report more problems of sexual dysfunctions in women with urinary incontinence, others report dyspareunia, low libido, and vaginal dryness more in patients with POP.^4^,^5^ However some studies report no difference in the sexual functions in women with POP or incontinence.^6^

Studies assessing the effect of surgery for POP and/or SUI on female sexual functions also give heterogeneous results. Reconstructive surgery for POP or SUI does not necessarily restore optimal sexual function; rather they might be unchanged, improved or worsened post- surgery.^7^,^8^ A systematic review and meta-analysis of 674 citations, assessing the impact of native tissue repair for pelvic organ prolapse on sexual function suggest significant improvement in sexual function and dyspareunia after native tissue for prolapse.^9^

Overall, the effect of these disorders on women’s sexual functions has been poorly studied, with more efforts focusing on cure of POP and incontinence, rather than on sexual functions. Furthermore, most of the studies have only assessed dyspareunia without using validated questionnaires, casting doubt on the validity of findings. We aimed to evaluate effects of urinary incontinence (UI) and/or POP on female sexual functions using a validated questionnaire. We hypothesized that there is no significant difference in sexual functions after surgery for POP and/or SUI.

The objective of this study was to assess various domains of female sexual functions in patients before and after reconstructive surgery for pelvic organ prolapse and UI by using FSFI questionnaire.

## METHODS

We conducted a quasi-experimental pre-post study at the Aga Khan University Hospital, Karachi, from January 1^st^ 2019 to December 31^st^ 2019 including sexually active females of age 25-65 years. A sample size of 126 was calculated using WHO sample size calculator, keeping proportion of women who were sexually active before surgery 82% and after surgery 89%, alpha 5%, and power 80%.^10^ Sampling technique was non-purposive consecutive sampling. We included all women attending the Urogynecology clinics with clinically diagnosed POP and/or SUI for at least three months duration, planned for reconstructive surgery and/or surgery for SUI. Women unwilling to participate, those with incomplete questionnaire and with neurological deficit or psychiatric condition were excluded from the study. After written informed consent, all women filled the FSFI questionnaire both in English and Urdu before surgery and 6, 12 and 18 months postoperatively. Female Sexual Dysfunction (FSD) was defined as a disorder in any of the sexual functions including sexual desire, arousal, orgasm, and/or pain causing personal distress. The FSFI questionnaire is a self –reported 19 questions scale. It asseses key dimensions of female sexual function in the last four weeks and has six domains: desire, arousal, lubrication, orgasm, satisfaction and pain.^11^ Weigel has described cross-validation and developed clinical cut-off scores for patients with and without sexual dysfunctions.^12^

All patients were evaluated by a detailed urogynecological history and validated questionnaires like Urogenital Distress Inventory (UDI-6) and the Incontinence Impact on Quality of Life (IIQ-7) Questionnaires. Data was collected for demographics including age, parity, BMI and duration of stable relationship. Urogynecological examination was performed using standard POP-Q staging. A pelvic ultrasound was done to exclude uterine or ovarian masses. Finally patients with suspected neurogenic bladder underwent urodynamic assessment. Continuous variables were described in terms of mean and standard deviation. Categorical variables were described in terms of frequency and percentages. Frequency of FSD and its various domains were recorded as per FSFI questionnaire. Patients were grouped into either having sexual dysfunction (FSFI<27) or no sexual dysfunction (FSFI≥27) as suggested by Wiegel et al.^12^ Data was computed using SPSS 21. Paired t-test was applied to see the difference in sexual functions before and after the surgery. P-value of <0.05 was considered significant.

### Ethical approval

Ethical approval for this study was taken from institutional Ethical Review Committee (Ref: ERC-5155-Obs-ERC -17, Dated: June-27-2018).

## RESULTS

A total of 126 patients presenting with POP and/or SUI with associated sexual dysfunctions, who underwent surgery for POP and/or SUI were included in the study. Basic demographics are shown in Table-I. Vaginal hysterectomy (VH), pelvic floor repair and mid-urethral sling (MUS) surgery was performed in 31/126 patients while 55/126 patients underwent VH with pelvic floor repair and 12 women had pelvic floor repair alone. Eighteen patients had MUS operation alone for SUI and 10 patients who opted for fertility preserving surgery had uterine suspension surgery for prolapse.

**Table-I:** Shows basic demographics.

Variables	SUI / POP group
Age (years) Mean ± standard deviation; range	51.6 ± 10.9; (23-65)
Parity Mean ± standard deviation; range	4 ± 1.6; (1-9)
BMI (kg/m2) Mean ± standard deviation; range	29 ± 3.36; (22-39)
***Mode of delivery n (%)***	
Nulliparous	10 (7.93%)
Vaginal delivery	92 (73%)
C sections	24 (19%)
***Menopausal status n (%)***	
Premenopausal	57 (45.2%)
Post-menopausal	69 (54.7%)
***POP stage n (%)***	
Stage 1-2	70 (55.55%)
Stage 3-4	56 (44.4%)
POP with SUI	31 (24.60%)
SUI alone	18 (14.28%)

BMI: Body Mass Index, POP: Pelvic Organ Prolapse, SUI: Stress Urinary Incontinence.

Changes in total and individual domain of FSFI questionnaire scores are shown in Table-II. The data suggests that there is a statistically significant difference with p-value <0.01 in total and individual scores of each domain of FSFI before and after surgery. Overall, the FSFI score evaluation and calculation showed the improvement in sexual function of patients with POP and SUI surgeries from a mean of 18.5 before surgery to 20.8 after surgery.

**Table-II:** Mean FSFI Domain Scores before and after surgery.

Parameters	Before surgery	After surgery	p-value
Arousal	3.18±1.3	3.50±1.4	< 0.01
Desire	3.20±1.2	3.50±1.3	<0.01
Lubrication	3.16±1.3	3.50±1.4	<0.01
Orgasm	3.10±1.3	3.50±1.4	<0.01
Satisfaction	3.20±1.2	3.50±1.4	<0.01
Pain	3.10±1.2	3.50±1.4	< 0.01

Total	18.57±7.1	20.8±7.8	<0.01

Data are presented as mean ± standard deviation, Individual FSFI scores before and after surgery is compared using Paired t-test, the total FSF scores before and after surgery are compared using ANOVA.

Data are presented as mean ± standard deviation, Individual FSFI scores before and after surgery is compared using Paired t-test, the total FSF scores before and after surgery are compared using ANOVA.

## DISCUSSION

In the present study there was a statically significant improvement in total and all individual domain scores for desire, arousal and orgasm, satisfaction and lubrication after surgery for POP and/or SUI.

There are a number of studies addressing the changes in sexual functions after surgeries for POP and SUI with varying outcomes.^13^ We used FSFI for assessing the impact of surgery on sexual functions although there are many other questionnaires available, which are used in many studies. A prospective, multi-center cohort study including 355 patients who underwent vaginal surgery with traditional methods for grade ≥ II, symptomatic POP, showed improvement in sexual functions assessed by Pelvic Organ Prolapse/ Urinary Incontinence Sexual Questionnaire (PISQ-IR) before and 12 months after surgery for POP.^14^ A prospective randomized trial of 78 patients, evaluating sexual function in women before and after VH for uterine prolapse of stage 2 or higher, reported improved anatomical and sexual function postoperatively. However, it was observed that VH might have negative impact on sexual function if new-onset or worsening dyspareunia or incontinence develops postoperatively.^15^

We had thirty-one patients who underwent concomitant surgery for POP and SUI and showed improvement in FSFI scores postoperatively. Similar results were observed in a prospective study of 159 women, where the use of transobturator tape (TOT) was associated with decrease in coital incontinence and significant improvement in FSFI desire and total scores with no significant difference in patients undergoing TOT and concomitant surgery group except for the worsening of lubrication.^16^

A study of 247 women by Sarreau et al. who underwent MUS for urinary incontinence, 81.8% found an overall improvement in quality of life after one year, assessed using the Patient Global Impression and Improvement (PGI-I) while 31.4% reported improvement in intercourse satisfaction.^17^ Improvement in sexual function after concomitant surgery for POP/SUI is mainly attributed to improvement in UI and leak accident. In contrast, Kim et al.^18^ found no difference in overall sexual function or in the individual FSFI domain scores using the FSFI in 47 women who underwent a TOT or retropubic MUS for urodynamic stress incontinence (USI).

We performed surgical repair of POP using native tissue and polypropylene MUS were used to treat SUI. The use of mesh has now become debatable but a recent study assessing the effect of transvaginal mesh surgery (TVM) on sexual activity and Quality of Life (QOL) of 237 Japanese women with Pelvic Organ Prolapse revealed significant improvement in QOL and overall scores for sexual function and arousal after surgery.^19^ Similar improvement was observed in other studies assessing female sexual functions, using FSFI after surgery for POP.^20^,^21^

The current study had 54% postmenopausal women and improvement in sexual function occurred regardless menopause. This is in contrast with a study by Long et al. who assessed the effect of TVM surgery on sexual function of both pre and postmenopausal women using FSFI. They revealed that despite an effective anatomical restoration of POP, TVM surgery was associated with worsening of individual and total domains of FSFI in premenopausal women postoperatively, with significantly higher rate of deteriorated dyspareunia than postmenopausal women.^22^

In our study, all domain of FSFI showed significant improvement after surgery in contrast to a study by Hoda and Kim et al. who found no significant change in orgasm function of women with anterior or posterior repair for prolapse.^18^,^23^

The strength of our study is that it involves the evaluation of sexual functions using a validated FSFI questionnaire both before and after surgery and prospective collection of data. Furthermore, the same surgeon operated all patients, thus reducing bias. No patient was lost to follow during the course of study. Our research highlights a very sensitive topic in developing countries where sexual functions are under reported.

### Limitation of the Study

It includes inability to perform a power study because of different surgical procedures in different patients and its small sample size. This is a single center-based study and cannot depict the change in trends locally seen. Despite all these limitations, the present study demonstrates that surgery for POP and SUI results in overall improvement in sexual functions in patients who underwent surgery for POP and/or SUI.

## CONCLUSIONS

This study shows improvement in overall sexual functions and individual domain scores of sexual desire, arousal, lubrication, satisfaction and orgasm after surgery for POP and/or SUI. This improvement is mostly due to post-surgical correction of the female anatomy. The overall improvement in sexual functions reveals that these surgeries are helpful in alleviating the sexual dysfunction that a woman experiences due to POP and SUI.

### Authors’ Contribution:

**SA:** Conceived the idea, study design, manuscript preparation and drafting.

**RMR:** Editing, review and final approval of manuscript. Responsible for integrity and accuracy of the work.

**HS:** Data collection and statistical analysis.
